# Recent Applications of Electricity

**Published:** 1902-11-22

**Authors:** George Parker

**Affiliations:** Physician Bristol General Hospital


### Hospital Clinics and Medical Progress.

### RECENT APPLICATIONS OF ELECTRICITY.

By George Parker, M.A., M.D.Camb., Physician Bristol General Hospital.

### III. Static Electricity.

Formerly the machines used for generating static electricity were of the frictional type, in which the charge was obtained by rubbers impinging upon the rotating plates. At present nearly all are of the influence type, of which the Holtz and Wimshurst machines are the most often used. They may be driven by hand or by motors, and must be protected from dust and damp especially in this climate. The Wimshurst is self-charging and some of the Holtz machines have a miniature Wimshurst in one corner to start them with a sufficient charge. Calcium chloride may be kept in dishes under the glass cover to ensure the dryness of the air and all the metallic poles and electrodes must be frequently polished and rubbed if successful working is to be attained.

Among the various methods of applying static electricity besides the direct spark we find—

1. Simple charging or static insulation.
2. Monell's potential alteration.
3. The breeze.
4. The friction spark or massage by a roller.
5. Morton's static induction.

In simple charging the patient is insulated on the platform and the positive pole is connected with his chair by a chain, while the other pole is grounded. When the machine is set in motion the patient becomes positively electrified, his hair stands up and a light surrounds him if the room is darkened. The condition may be continued for a quarter of an hour or longer. The general effects are refreshing, but, if the positive pole is grounded, the effects of the negative pole are said by some writers to be depressing. However, simple charging is a preliminary to all other treatment on the insulating platform.

In potential alteration the charge is suddenly removed without discomfort to the patient by bringing the grounded electrode near the positive knob and thus creating a short circuit by a spark from one to the other. If this is repeated several times rapidly the patient undergoes a frequent and a fairly vigorous change of potential. Similarly, when the patient is insulated the static breeze may be employed either by the indirect or direct method; that is the charge of electricity in the patient may be withdrawn through a conductor held too far away to permit sparking, and passed either through the earth and a grounded conductor to the opposite pole, or direct to that pole without going through the earth. The current is felt as a cool wind blowing towards the patient when the conductor is held anywhere between sparking distance and a yard off. The nearer it is brought the stronger are the effects, and when near the sparking point the sensation resembles that of a douche of hot water. The presence of clothing introduces other modifications; thus a hot pricking sensation may replace that of a cool breeze, though the therapeutic effects are the same. We may employ this method for the relief of pain, such as neuralgia or headache, or as a powerful stimulant, according to the distance at which the conductor is held, and the strength of our current. Electric massage is given by passing the roller electrode quickly over the clothing when showers of small sparks fly from it to the patient. The strength of the application is increased with the thickness of the clothing, and may be decreased by first partially withdrawing the charge from the patient by placing one's own foot on the platform.

Morton's static induction, as we have said before, is in a sense an application of high frequency currents. If two Leyden jars are fitted to a Wimshurst machine, and the outer coatings are connected, a secondary circuit is formed, and at the same time the primary circuit across the spark gap is intensified. Instead of a direct connection between the two outer coatings, conducting wires and ordinary moistened electrodes are used to convey the secondary current to a patient, the force being regulated by altering the distance of the spark gap on the primary circuit, and by using larger or smaller jars. Thus we can obtain a current which is almost painless and which produces muscular contractions of a strong type. This current is applied to a patient just like one from the ordinary galvanic or Faradic battery.

Lewis Jones[7] lays down that in using this current we must adjust the sparking distance between the discharging electrodes on the machine before commencing the treatment. From an eighth to a quarter of an inch is generally sufficient; indeed, with a quarter of an inch spark the treatment is severe.

The therapeutic effects of static electricity have been the subject of many ill-founded claims and rash theories; but, as in other forms of electrical treatment, there is a residuum of real value. We need not even despise its psychical use as a means of suggestion in hysterical conditions, but there are direct physical results of more importance. It may be applied either in a general or localised manner. Static insulation or simple charging is a type of the one, and Morton's induced current and the breeze are instances of the other. In general treatment the nutrition of patients is improved, their appetite and sleep return, the output of urea is increased, while uric acid is lessened. General applications have also given very good results in neurasthenia, hysteria, some forms of melancholia, obesity, and dysmenorrhœa, from their stimulating or tonic effect. They have this advantage over the electric bath, that they do not necessitate the removal of the clothing.

Localised treatment by Morton's currents directed to a motor point or nerve trunk brings into play damaged muscles and is of great value in various forms of paralysis, muscular rheumatism, and perhaps in neuralgias, though Jacoby throws doubt on its effect in the last case. The perfect manner in which it can be applied to a given spot only, and its force regulated, are one of its great advantages. The breeze is valuable chiefly as a sedative in pain, while sparking is used as a stimulant or counter-irritant. Indeed these three methods may respectively be compared to sinusoidal, galvanic, and Faradic treatment, though certain differences exist in each case.

[3] Electro Therapy, vol. 1, p. 186. [5] Therapeutic Electricity.

[4] Electro Therapy, vol. 2, p. 151. [5] Med. Electricity, ed. 3, p. 190.

[6] Lancet, Oct. 18, 1902. [7] Med. Electricity, ed. 3, p. 177.

